# Interoception, contemplative practice, and health

**DOI:** 10.3389/fpsyg.2015.00763

**Published:** 2015-06-09

**Authors:** Norman Farb, Jennifer Daubenmier, Cynthia J. Price, Tim Gard, Catherine Kerr, Barnaby D. Dunn, Anne Carolyn Klein, Martin P. Paulus, Wolf E. Mehling

**Affiliations:** ^1^University of Toronto Mississauga, Mississauga, ONCanada; ^2^University of California San Francisco, San Francisco, CAUSA; ^3^University of Washington, Seattle, WAUSA; ^4^Maastricht University, MaastrichtNetherlands; ^5^Brown University, Providence, RIUSA; ^6^University of Exeter, ExeterUK; ^7^Rice University, Houston, TXUSA; ^8^University of California San Diego, La Jolla, CAUSA

**Keywords:** interoception, contemplative practice, meditation, body awareness, mindfulness, yoga, mind–body therapies

## Abstract

Interoception can be broadly defined as the sense of signals originating within the body. As such, interoception is critical for our sense of embodiment, motivation, and well-being. And yet, despite its importance, interoception remains poorly understood within modern science. This paper reviews interdisciplinary perspectives on interoception, with the goal of presenting a unified perspective from diverse fields such as neuroscience, clinical practice, and contemplative studies. It is hoped that this integrative effort will advance our understanding of how interoception determines well-being, and identify the central challenges to such understanding. To this end, we introduce an expanded taxonomy of interoceptive processes, arguing that many of these processes can be understood through an emerging predictive coding model for mind–body integration. The model, which describes the tension between expected and felt body sensation, parallels contemplative theories, and implicates interoception in a variety of affective and psychosomatic disorders. We conclude that maladaptive construal of bodily sensations may lie at the heart of many contemporary maladies, and that contemplative practices may attenuate these interpretative biases, restoring a person’s sense of presence and agency in the world.

## Introduction

### Introducing Interoception

Interoception is the process of receiving, accessing and appraising internal bodily signals. Maintaining desired physiological states is critical for an organism’s survival, and so interoception is a powerful motivator of behavior in the pursuit of these states (Craig, 2002, 2009). While interoceptive awareness has been more narrowly defined as the representation of afferent body sensations (Craig, 2003; Critchley et al., 2004), broader definitions cast interoception as a multi-dimensional construct that also takes into account how people attend to, appraise and respond to these sensations (Vaitl, 1996; Cameron, 2001; Verdejo-Garcia et al., 2012). Interoception is supported by an increasingly well-specified neuro-anatomical pathway with dedicated representation cortices akin to the five external senses (Craig, 2002; Critchley et al., 2004; Critchley and Harrison, 2013), although the nature of such representation is decidedly more mysterious and in need of empirical investigation.

Interoception is an iterative process, requiring the interplay between perception of body states and cognitive appraisal of these states to inform response selection. Afferent sensory signals continuously interact with higher order cognitive representations of goals, history, and environment, informing emotional experience and motivating regulatory behavior (Craig, 2009). Together, these iterations create a sense of self laden with motivational context (Damasio, 2003b). Such context may then inform a person’s approach or avoidance tendencies, and thus these interactions hold important implications for well-being (Beck, 2008). Conversely, life experience impinges upon interoception: the perceived availability and relevance of body representations are moderated by factors such as attentional cues (Ainley et al., 2012), contemplative training such as meditation, yoga, tai chi, etc. (Bornemann et al., 2014), and age (Khalsa et al., 2009). Understanding how interoceptive processing is shaped by experience is therefore important for efforts to cultivate well-being and stress resilience.

### Interoception and Well-Being

One reason that interoception, a perceptual capacity, is introduced in relation to well-being is that it is thought to be intimately connected to self-regulation, having likely evolved to help organisms maintain homeostasis (Paulus, 2007; Herbert and Pollatos, 2012; Craig, 2013; Gu and Fitzgerald, 2014). In modern life, emotionally valenced body signals are also thought to contribute to broader mood states that support emotional balance (Paulus and Stein, 2006; Craig, 2007; Seth, 2013). To the extent that a person is sensitive to interoceptive signals, such signals guide decision making (Dunn et al., 2010). And yet, high sensitivity is not without its price: when body sensation is irregular, as for individuals with joint hypermobility, greater interoceptive sensation may also contribute to feelings of anxiety (Mallorquí-Bagué et al., 2014). Thus, interoceptive sensitivity may either contribute or detract from well-being, suggesting a need for guidance in regulating salient visceral signals.

Paralleling the findings of modern secular clinical science, contemplative science suggests that reflection on interoceptive processes is important for adaptive behavior (Wallace, 2007), disrupting overlearned perceptual, and interpretive habits formed throughout cognitive development and aligning behavior with higher-order intentions (Vago and Silbersweig, 2012). As the embodied self is more fully realized through awareness of ongoing interoceptive interactions, two complimentary senses emerge: *presence,* one’s connection to the moment, and *agency,* one’s ability to effect change, which are both foundational in determining a person’s sense of well-being (Seth et al., 2011). To chart interoception’s salutary relevance, research must therefore consider the integration of both early sensory and later affective discriminations into a broader motivational context.

### Motivation for the Present Paper

Over the past decade, the mechanisms of interoceptive awareness, the importance of interoceptive cues for physical and psychological health, and the cultivation of healthy interoceptive habits through contemplative practices have become topics of active investigation. However, insights from these three domains are rarely integrated, impeding the progression of understanding their collective significance. In April 2013, the authors met to discuss interoception across a variety of perspectives – including neuroscience, social and clinical psychology, complementary and alternative care, medicine, Buddhist and contemplative studies, and a wealth of personal experiences with contemplative practices. Our goal is to better explain how interoceptive information can be revealed and masked from conscious awareness, how our appraisals of a given level of interoceptive awareness help to determine well-being, and what role contemplative practices may play in these processes.

Here, we attempt to integrate contemporary interoceptive theory from these diverse perspectives, in the hope of better specifying interoceptive constructs and identifying next steps for advancing the field. First, we define interoception from scientific and contemplative perspectives, focusing on a predictive coding model of interoceptive representation that is growing in popularity within the scientific community. Second, we discuss the role of interoception for physical and psychological well-being, with particular regard for emerging clinical evidence. Third, we explore how contemplative practices may interact with interoceptive processes to enhance well-being in oneself and connection to others. Finally, we summarize outstanding issues in the study of interoception and offer recommendations for future research.

## Interoception in Psychology, Neuroscience, and Contemplative Traditions

### Toward a Science of Interoception

The scientific study of brain, mind, and body provides a framework for objectively modeling interoception. Neuroanatomical studies have identified pathways that support and modulate interoceptive representations, and as such, may be measurably impacted by psychopathology or contemplative practice. This framework is comprised of peripheral receptors, C-fiber afferents, spinothalamic projections, specific thalamic nuclei, posterior and anterior insula as the limbic sensory cortex, and anterior cingulate cortex (ACC) as the limbic motor cortex (Vaitl, 1996; Bush et al., 2000; Vuilleumier, 2005; Critchley and Harrison, 2013). In some cases, definitions of interoception extend beyond visceral afferents (Sherrington, 1906) to include afferents from other deep body receptors, such as C-tactile somatic receptors of the skin that are associated with internal feelings of warmth or pleasure from gentle touch (Björnsdotter et al., 2009), circulating chemicals, proprioceptive inputs, and unexpected or yet unknown sites, such as recently discovered photoreceptor cells within the eyes (Lucas, 2013).

The anatomical pathways for interoception are well-specified, detailing the connections between sensory receptors, spinal cord, brainstem, and brain (Craig, 2002). Neurotransmitter concentrations in the insula and ACC in particular have been associated with subjective interoceptive awareness and subjective well-being (Ernst et al., 2014; Wiebking et al., 2014). However, how such neural representations are constructed and the mechanisms by which they influence cognition remain unclear. Most investigations have focused narrowly on interoceptive awareness, which may only be one aspect of interoceptive processing. Interrelated but distinct constructs such as interoceptive attention tendency, sensitivity, coherence between physiology and subjective experience, accuracy, and regulatory habits may all emerge as important properties of information propagation and integration throughout interoceptive networks (**Box 1**).

**Box 1. A taxonomy of interoception terms.**

Interoception has been generally construed as the representation of the individual’s body at a particular point in time (Craig, 2002), but accounts differ on how interoceptive capacity should be measured. Similar to a recently proposed model (Garfinkel and Critchley, 2013), we suggest several criteria to supplement the measurement of interoception:

• Interoceptive *awareness*, such as the sense of one’s heart beating, is the most common measure of interoception, and is usually operationalized in terms of the reportability of interoceptive signals. However, awareness may be a limited criterion, as interoceptive processes may operate implicitly, such as thermoregulation promoting shivering even when one is asleep (Craig, 2003).

• *Coherence* between physiological and subjective states measures the degree to which objectively observable interoceptive signals manifest in reportable experience. For example, low blood sugar (hypoglycemia) is characterized both by interoceptive signals (Herbert et al., 2012a) and irritability (Matthew et al., 1997). However, awareness of low blood sugar is not necessary for it to promote irritability (Taylor and Rachman, 1988; Matthew et al., 1997). Coherence appears to vary widely among individuals, even though both are impacted by environmental stressors (Sze et al., 2010).

• *Attention tendency* refers to whether a person habitually attends to particular interoceptive signals, potentially inhibiting or ignoring others (Pollatos et al., 2005). It can also be expressed as whether a person attends more to interoceptive than exteroceptive sources of information.

• *Sensitivity* refers to the minimum threshold for detecting interoceptive signal change (Holzl et al., 1996); sensitivity may operate at multiple levels of the representational system, culminating in access to conscious awareness. Although not often used in interoception research, specificity is the common counterpart to sensitivity in signal detection theory (Abdi, 2007), the ability to reject competing signals from being classified as interoceptive afferent signals.

• *Accuracy* is perhaps the most commonly investigated measure of interoception, and refers to the ability to reliably discriminate interoceptive signals from noise or competing signals (Vaitl, 1996), and between different levels of signal intensity (Daubenmier et al., 2013). It appears that accuracy varies widely between individuals (Critchley et al., 2004; Ceunen et al., 2013), but may improve with focused training (Brener, 1977; Daubenmier et al., 2013; Mirams et al., 2013). Accuracy is usually thought to be a function of sensitivity and specificity.

• *Sensibility* refers to an individual’s personal account of how they experience internal sensations, including a confidence estimate of one’s own interoceptive ability and feelings of engagement during interoception (Garfinkel et al., 2015). Sensibility is often gaged using interviews and/or questionnaires, frequently the Porges Body Perception Questionnaire (Porges, 1993) is applied. The usefulness of this instrument for interoception, however, has been questioned as it appears to primarily serve as a proxy measure for anxiety-related symptoms (Mehling et al., 2009). The Scale of Body Connection (Price and Thompson, 2007) and the Multidimensional Assessment of Interoceptive Awareness (MAIA; Mehling et al., 2012) were created in part to help extend interoceptive assessment.

• *Regulation* refers to how well a person can match an interoceptive signal to his or her desired state. Regulation can involve shaping either the signal or the desire. For example, regulation could shape interoceptive signals to meet goals through reappraisal, suppression, or distraction, techniques often cited in modern scientific models of emotion regulation (Gross, 2002). However, regulation could also follow more contemplative traditions, intentionally accepting and examining such signals with curiosity, a strategy that encourages shifts in interoceptive experience without attempting to control unexpected interoceptive signals or create desirable ones. Concurrent measurement of these constructs may inform a more complete model of interoception, its relationship to well-being, and the effects of contemplative training

It is important to note that these constructs have varied definitions across the literature. Sensitivity and accuracy are combined as interoceptive accuracy in a recent interoception model (Garfinkel et al., 2015), measured as the performance on objective behavioral tests such as the heartbeat detection task, and clearly distinguished from interoceptive awareness and interoceptive sensibility. In the Garfinkel et al. (2015, p. 65) model, interoceptive awareness has been operationalized as “metacognitive awareness of interoceptive accuracy, e.g. confidence-accuracy correspondence.” However, the term metacognitive awareness is also a term commonly used in contemplative practice pointing to the possibility of taking awareness itself and the process of thinking as an object of attention and has been defined elsewhere as the ability to reflect or be aware of ongoing thought or mental states (Smallwood and Schooler, 2006; Epel et al., 2009). There, it has been defined as the ability to experience negative thoughts and feelings as mental events that pass through the mind, rather than as a part of the self (Herbert and Forman, 2011). We would therefore avoid using the term “metacognitive awareness” to mean confidence–accuracy correspondence, preferring the term coherence instead. Thus, the terms listed above represent an attempt to bring a common taxonomy to our discussion, but should not be read as universally definitive in this developing field of inquiry.

Adding to the complexity, multiple interoceptive sub-systems operate concurrently, and may each afford different capacities and tendencies. There is some evidence for general, multimodal interoceptive capacity: heartbeat detection accuracy is correlated with gastric sensitivity (Herbert et al., 2012b), and sometimes with pain sensitivity in some studies (Pollatos et al., 2012), but not with others (Werner et al., 2009). Furthermore, the relationship between interoceptive constructs across domains such as the heartbeat, blood glucose, respiration, temperature and other modalities is largely unknown. This gap in understanding may be due to the fact that most interoception studies gage accuracy within a single modality, and do not measure related constructs such as attention habits, regulation ability, or sensitivity to signal change, all of which converge to support interoceptive awareness (Ceunen et al., 2013).

Acknowledgment of interoception’s multimodal and multifaceted nature is important, because consideration of these factors in isolation can lead to spurious inferences. For example, knowledge that meditators have strong interoceptive attention tendencies might lead one to expect them also to possess superior accuracy in heartbeat detection, an idea contradicted by recent research on this topic (Khalsa et al., 2008; Parkin et al., 2013). The benefits of interoceptive attention in a particular domain such as breath or body monitoring therefore seem to be independent from a domain-general enhancement of interoceptive accuracy. Instead, meditation practices appears to promote changes in a more specific subset of interoceptive capacities and tendencies, accompanied by non-interoceptive factors such as changes in intentions or goal-orientations unique to each system of practice (Bornemann et al., 2014).

Just as capacity may vary across interoceptive modalities, interoceptive constructs may also vary independently within a given modality. For example, sensitivity may be high without commensurate accuracy or regulation ability, such as with uncontrollable interoceptive ‘false alarms’ often observed in anxiety disorders (Paulus and Stein, 2006; Domschke et al., 2010). For this reason, rather than arguing for the primacy of any of one interoceptive construct, the present discussion uses the term interoception to describe general processes of perceiving body states, while also acknowledging that a variety of contextual factors influence specific interoceptive capacities. A huge amount of research is needed to begin to catalog the relationships between specific capacities and modalities, let alone their plasticity and effects on well-being.

### Integrating Contemplative Insights

If we could establish a working model of interoception that accounts for differences in how interoceptive signals are represented and managed, it is likely that some sub-systems would be more relevant for well-being than others. It is at this point that contemplative science and paradigms of cognitive processing may be extremely helpful, implicating particular interoceptive processes, addressing how interoception ideally functions, and how such function is shaped by experience. In particular, contemplative accounts have much to say on the distinction between maladaptive interoceptive conditioning that may lead to disorders, and adaptive learning that is purportedly engendered by contemplative practices.

Here, we refer to contemplative practice in the broadest sense, i.e., traditions of first-person reflection upon or cultivation of specific modes of experience, and focus on those practices that explicitly involve interoceptive awareness, including types of meditation and mindfulness-based approaches that allocate attention to body sensations (e.g., the breath), or to specific areas of the body (e.g., the center of the abdomen; Kabat-Zinn, 1982), and yoga, tai chi, and other mind-body practices performed in or outside of an explicit spiritual context (Baer, 2003). A wealth of other body-oriented healing and psychotherapy methods may also fall into this classification, such as Dialectical–Behavioral Therapy (Linehan et al., 1999), Acceptance and Commitment Therapy (Hayes et al., 1999), and also body-mind approaches such as Feldenkrais (Buchanan and Ulrich, 2001), Alexander method (Woodman and Moore, 2012), Focusing (Gendlin, 2012), Rosen work (Fogel, 2009), Hakomi (Kurtz, 1997), Sensory Awareness (Selver et al., 2007), Somatic Experiencing (Payne et al., 2015), Breath Therapy (Mehling, 2001), Holotropic Breathwork (Grof and Grof, 2010), and Mindful Awareness in Body-oriented Therapy (MABT; Price, 2005). We discuss primarily practices derived from Asian contemplative traditions, as these have undergone the most research and been the focus for the largest effort to translate contemplative concepts into modern scientific terms. This is not meant to exclude other contemplative traditions, and we hope future research will be extended into other such areas.

Classical contemplative practices such as mindfulness and equanimity seem to speak to the issues of how interoceptive signals are integrated into a complex representation of self and the world beyond, and have models of how they affect health and well-being. There are, however, several outstanding challenges to realizing the potential benefits of these classical traditions in secular modern society. The first issue is the lack of equivalence between traditional contemplative and modern secular practices: although mindfulness has found a strong representation in secular clinical therapies and sciences, it is disputed whether this translation remains faithful to its classical sources (Christopher et al., 2009; Grossman, 2011), and little attention has been given to the diversity of interpretations of mindfulness within the Buddhist tradition itself (Williams and Kabat-Zinn, 2011), which may present competing models for interoceptive representation and function. Second, while both clinical science and contemplative traditions share the goal of reducing human suffering, they differ in scope. In modern secular contexts, practice goals are largely pragmatic, aimed at reducing affective symptoms and improving daily function. By contrast, classical traditions tend to orient toward more extensive change, seeking to generate insight into the fundamental nature of reality, with the intention of liberating individuals from their conditioned states (Grossman and Van Dam, 2011). It is uncertain whether current appropriations of classical contemplative practices are sufficient to promote their historically lauded benefits. Given emerging evidence of contemplative practices’ efficacies in secular contexts, there is reason for optimism that at least some beneficial elements have been successfully translated (Farb, 2014). However, our understanding of these practices’ true mechanisms of action has lagged behind the scientific validation of their efficacies. There may be central constructs in classical contemplative theory that have yet to be operationalized in scientific discourse, let alone empirically studied.

Despite the great heterogeneity among contemplative traditions, we may begin by selecting a common concept that may contribute to scientific discourse: that of the ‘subtle body’. Contemplative practices such as mindfulness are traditionally grounded in traditions, epistemologies, and medical treatises that articulate holistic rather than dualistic models of body and mind (Mehling et al., 2011). Such traditional sources each have their own distinct theories of the psycho-physical complex and invoke concepts of subtle body structures and ‘currents’ flowing through those structures (Samuel, 2008; Klein, 2013). These structures and ‘flows’ are amenable to influencing and being influenced by the mind, emotions, posture, and the condition of the grosser (physical) body. Their presence is indicated through awareness of a rich array of internal body sensations and a long phenomenological history of sensate processes that relate events occurring in the outside world to the experience within an individual. Exploring these somatic sensations, their sources, and their modulation has been an important focus of Tibetan, Chinese, and Indian medicine, represented in anatomical maps of channels, meridians, and ‘energy centers’ (chakras: Sanskrit) through which ‘subtle energies’ pass known as lung/ch’i/prāṇa, respectively (Loizzo et al., 2009; Klein, 2014). Every mental event – that is, all states of consciousness- are said to ride the ‘steed of wind’ or ‘energy’ currents. It is currently unclear how these conceptualizations map onto scientific approaches of interoception. However, these concepts do suggest that attention to embodied experience is significant for self-representation and well-being, and therefore supports the more general hypothesis that over-dependence on top–down, or merely conceptual (in contrast to sensory) awareness significantly limits a human being’s potential for relating to self, others, and the world.

### Integration Challenges

Asian classical traditions have a rich history of describing the integration of varied interoceptive signals into a common representation, a phenomenon often referred to as the ‘subtle body’ (Samuel, 2008; Klein, 2013). The subtlety of this body has to do with its typically functioning outside the horizon of ordinary consciousness. However, as already indicated, as the ‘steed’ or support of consciousness, it impacts perception significantly. We suggest that scientific discourse is just beginning to address this question of integrated representation, creating new possibilities for understanding what differentiates wholesome from maladaptive integration states. And yet, there are few empirical accounts of such integration, and so the potential to operationalize fundamental constructs from these traditions and practices is largely unknown. For example, many contemplative traditions, and many practitioners, speak of changes in the ‘flow of energy,’ resulting in feelings of lightness or heat, but it is largely unknown whether modern psychophysiological research methods can detect such changes (Loizzo et al., 2009; Kozhevnikov et al., 2013). Physiological arousal itself is inherently ambiguous and highly constrained by cognitive appraisal (Blascovich and Tomaka, 1996), and the science of how interoceptive appraisals translate into distinct emotional experiences is inexact at best (Wilson-Mendenhall et al., 2013). Given this uncertainty, we might begin by searching for the training-related changes described in classical contemplative theories of interoceptive integration. Such descriptions offer a rich set of testable hypotheses for filling this explanatory gap between physiology and experience. Inclusion of first-person experiences, particularly from people with extensive training in directing and reporting on interoceptive attention, may be an important step in developing more comprehensive models of interoception (Gallagher, 1997; Lutz and Thompson, 2003).

As an example of such hypothesis testing, we might consider a classical description of the first steps in mindfulness training. Classical Buddhist texts, such as the fifth century *Path of Purification,* describe the primary goal of early mindfulness training is to develop stable awareness of momentary sensation, distinguishing it from awareness of conceptual thought (Buddhaghosa, 2010). Recent scientific investigation suggests that the conceptualization of an experience, which may include detailed description and analysis of events that are associated with the experience and shaped by our collective cultural socialization, and the actual felt, immediate experience itself, which may arise in the body spontaneously, activate dissociable neural networks, and that the strength of this dissociation is indeed sensitive to mindfulness training (Farb et al., 2007). In this way, contemplative traditions provide a ground for distinction that is ripe for translation into objective, neuroscientific terms. Representation of the momentary, sensory self is relevant to practices of compassion and insight, as well to the classic mindfulness practices that undergird them.

Learning to attend to visceral, momentary sensation is only one step of many in the path to well-being, and likewise a neural distinction between immediate interoceptive and conceptual processing is only an initial step in the development of a scientific model. As will be discussed, access to visceral sensations may be helpful or harmful to a person depending on how such awareness is understood. Under the right conditions, contemplative practices may have therapeutic impact on health and well-being (Farb et al., 2012a), as has been shown in experiential psychotherapy research for some time (for a review, see Hendricks, 2001). On the other hand, interoceptive signals may also be catastrophized in panic and related disorders. Understanding how to skillfully relate to interoceptive sensations, and under what circumstances they should be attended to, is therefore a central question for the study of interoceptive training for well-being. To progress from a general description of interoceptive processing toward characterizing the wholesomeness of such processing requires a greater level of theoretical complexity, the beginnings to which we will attempt to address.

### Regulation through Simulation

A scientific model of interoception requires some description of how diffuse sensory signals become integrated into a holistic representation of the body, one that is amenable to integration with higher-order cognition. The concept of a *simulation map* provides one way to characterize such integration. An analog of the contemplative subtle body, the simulation map is also known as an “as–if” representation, a neural representation of the body, a relatively stable abstraction of rapidly fluctuating sensory events (Damasio, 2003a; Seth and Critchley, 2013). From a computational perspective, the simulation map is the ongoing selection of encoded body states into a working memory buffer that serves as the best approximation of the body’s current state as predicted by these prior states. The simulation map is layered, with lowest layers being closest to raw sensory afferents from the body, and higher layers representing the aggregation of information at these lower layers into representations that may be accessible to consciousness. As such, the simulation map affords executive brain areas with relatively stable sensory representations from which to interpret experience and coordinate responses. The concept of a simulation map provides a rich canvas on which to observe the nuances and variability in the cognitive representation of interoceptive signals, and furthermore suggests how such representations may be altered, both through the voluntary deployment of attention, and through the relatively involuntary processes of attentional conditioning across the lifespan, such as sensory degradation in aging (Baltes and Lindenberger, 1997).

It is important to stress that the simulation map is not identical to current sensation, but is rather an abstraction from recent sensory experience. Current sensation refers to the current set of sensory inputs arriving at primary interoceptive representation areas of the brain, most likely including the nucleus of the solitary tract, thalamus, posterior insula, and somatosensory cortices (Craig, 2002; Critchley and Harrison, 2013). The simulation map on the other hand is a filtered and integrated representation of these sensory afferents- it is, in effect, an interpreted signal. When considering regulatory motivation, a central question is the degree to which unexpected sensations are viewed as acceptable, as opposed to problematic, deviations from one’s expected body state.

Like many neural representations, the contents of the simulation map may not be fully accessible to consciousness. Of the many simulation layers, only a subset will be accessible to conscious experience. Thus, the conscious, *phenomenal map* may not be the same as the broader simulation map, although we view the simulation map as necessary for phenomenal bodily experience. Furthermore, there is large inter-individual variability among phenomenal maps as a function of goals, culture, personal experience, and possibly genetics and other contributing factors (Ferron, 1997; Altabe, 1998; Ma-Kellams et al., 2012; Maister and Tsakiris, 2014). These factors may combine to limit how the simulation map is consciously accessed, and a history of biased simulation map access may override momentary introspective efforts. Given that the simulation map may only be partially accessible to conscious introspection, it may be impractical to rely entirely on subjective reports to gage individual or cultural differences in simulation map composition. However, the simulation map may be tractably examined at the level of neural representation, serving as a substrate for a person’s sense of embodiment in the world. Through investigation of the simulation map, the visceral source of our sense of presence and our motivations toward action may be understood.

Neuroscience techniques have helped to move beyond subjective reports in modeling interoceptive simulation maps. By tracking changes in interoceptive signals and subjective body experiences during recordings of brain activity, researchers have begun to create rich models that distinguish between bottom–up sensory signals and top–down sensory predictions. For example, neuroimaging research suggests that attention to the body increases activity at the corresponding level of the spinal cord (Nejad, 2014), suggesting that interoceptive attention may operate even upon relatively distal aspects of the central nervous system. Within the brain, it appears that the middle insula integrates afferent interoceptive information with exteroceptive context into broader motivational space, but individuals differ in the degree to which such information propagates to the prefrontal cortex, and presumably conscious awareness, as a function of interoceptive practice (Singer et al., 2009; Farb et al., 2012b). In addition to neuroimaging, it should be noted that many other psychophysiological indicators of interoceptive processing show promise for revealing interoceptive processing in the absence of participant report, such as heartbeat-evoked potentials (Leopold and Schandry, 2001), respiratory-related potentials (Von Leupoldt et al., 2010), cardiac modulation of startle (Schulz et al., 2009), or EEG-ECG single trial covariation (Mueller et al., 2013). For example, Von Leupoldt et al. (2010) demonstrated that respiratory occlusion produced a reliable respiratory-related evoked potential measurable by EEG, which serves as an index of cortical tracking of interoceptive signals. Mueller et al. (2013) indexed interoceptive processing by the degree to which EEG responses to task feedback predicted subsequent heartbeat acceleration. While it is not our intention to describe all of such techniques in depth, such methods may provide objective indices of the impact of interoceptive signals at different levels of neural representation, such as the brainstem or cortex.

Perhaps the most important application of the simulation map is in explaining how visceral feeling promotes action. This explanatory construct is needed in both scientific and contemplative accounts of bodily health: analogous to the simulation map, contemplative traditions posit the existence of a subtle body, including various quasi-physical and delicate elements of the body that integrate and organize visceral feelings to provide a sense of well-being (Samuel, 2008). Across both scientific and contemplative traditions, motivational salience is afforded to simulation maps or subtle bodies in the form of emotional valence, with aversive or negative valence signaling a need to return to certain adaptive homeostatic ranges. The motivated process of achieving homeostasis, through physiological or behavioral change, has been dubbed *allostasis* (Sterling and Eyer, 1988).

In mammals, allostasis comprises many integrated functions and includes autonomic, neuroendocrine, and behavioral mechanisms. Much of allostasis occurs through autonomic self-regulatory physiology- it occurs internally and automatically. For example, we do not choose nor directly sense the dilation of blood vessels or pupils in response to changing luminance or emotional relevance, and yet such physiological accommodations occur continuously to promote homeostatic ends. A complete understanding of interoception doubtlessly requires the modeling of these allostatic reflexes, especially when their dysregulation acts as a source of suffering. Theories of early contemplative practice are agnostic to the ultimate accessibility of such responses; although direct experience of one’s visceral stress response and its triggering conditions are essential for the types of insights that will promote the liberation from motivation driven by conditioned expectations (Goleman, 2008; Hart, 2011). At some point, simulation map theories must account for how even subtle representations can lead to conscious insight that promotes behavior change.

The relationship of the simulation map to Asian (especially Indian and Tibetan) mappings of the ‘energy circuits’ within the body bears further investigation. At this stage, we note that, like the contents of the simulation map, many ‘energy flows’ within the body are not available to consciousness. However, these ‘energy flows’ are *always*, in traditional theorizing, intimately associated with consciousness. These ‘flows’ are capable of being brought to consciousness through training. As with the simulation map, these ‘energies’ seem formative of a person’s sense of embodiment, emotional orientation, agency, purpose, and sense of self-worth. Unlike the simulation model, perhaps, the mind-energy interaction is less dualistic than the visceral-cognitive dyad that so far seems central to characterization of the simulation map.

Leaving aside the question of how very subtle interoceptive afferents can be shaped to yield conscious insight, interoceptive signals motivate a wealth of unsubtle, overt behavior to satisfy allostatic concerns. Interoceptive dynamics are critical to understanding why identical stimuli can promote divergent behaviors. For example, a person will approach a heat source in a cold environment but avoid it when the ambient temperature is high. Conversely, an ice cube in one’s hand can feel pleasant during a hot summer day but aversive in winter. The meaning of the ice cube cannot be modeled based on the sense-perception of the ice cube alone, but rather the coolness of the ice is situated within a broader interoceptive milieu, one that reward or punishes cold sensation in response to thermoregulatory imperatives. Furthermore, allostatic cues can inform cognition and behavior at higher levels of representation- our trust in others is exaggerated by warm sensations and attenuated by cold sensations (Kang et al., 2011).

If an ice cube’s value is deeply influenced by allostatic concerns, a complete model of interoception must describe how such influence comes about. While acknowledging that many physiological perturbations are addressed unconsciously, through autonomic control of the internal milieu, conscious regulatory acts seem structured to resolve interactions between the body and its external environment (Gu and Fitzgerald, 2014). In this way, interoceptive signals motivate overt behavior that feeds back upon our physiology. It is also important to note that not all motivated behavior is allostatic: hedonic and pragmatic goals also have a large role to play, such as sensation-seeking to distract or regulate low mood (Taylor and Hamilton, 1997), or self-caffeinating in the face of fatigue (Lorist and Tops, 2003). Thus while homeostatic demands provide a convenient heuristic for predicting regulatory motivation, we must consider that many of our dominant drives sacrifice balance in the body to achieve other goals: riding roller-coasters, watching horror movies, and consuming spicy or sugary foods all fall into this category, in which extremes of arousal are intentionally provoked and enjoyed. Interoceptive regulation thus includes more than allostatic goals, but indeed any action intended to reshape the sensory signals that constitute the interoceptive simulation map. Rather than basing motivation on homeostasis, it may be represented more flexibly by how closely sensations match predicted or desired states.

### The Predictive Coding Model of Interoception

In recognition of the complexity of interoceptive processing, the “predictive coding” model of interoception has recently been introduced to the discourse on interoception (Seth et al., 2011; Limanowski and Blankenburg, 2013; Seth, 2013; Apps and Tsakiris, 2014). This model makes use of a final critical attribute of interoception, in that interoceptive processing regularly involves a comparison between immediate sensation and simulated past and future states. Comparison between observed and expected or desired states may then motivate behavior to resolve the discrepancy (Paulus and Stein, 2010; Seth, 2013). From an evolutionary perspective, awareness of interoceptive processes may promote adaptive behavior (Damasio and Carvalho, 2013), and body-focused contemplative practices may support such awareness (Mehling et al., 2011; Price et al., 2012b). We shall argue that the predictive coding model helps to operationalize many of the claims around the role of interoception in contemplative practice, particularly surrounding the deconditioning of maladaptive regulatory habits.

The idea that the simulation map can represent states distinct from one’s current interoceptive milieu suggests that comparisons are continuously being made within a given layer of the map, between the current sensation of the body, and the body as it is expected to be based upon past experience (the prior). The simulation is constructed through the consideration of both ongoing sensation and priors which contextualize these sensations. Each layer of the simulation map has its own set of priors, based on that layer’s experience with past sensory inputs and adjustments to these priors influenced by top–down expectancy signals. When a prior diverges from incoming sensation, it may update the simulation at that layer, creating a posterior probability that serves as a descending input for lower layers of the simulation map. For example, a person may go outside and be surprised by unexpectedly hot weather. Such surprise may begin at lower layers of the simulation map, promoting a response that is largely unconscious and automatic, targeting local physiology, such as the dilation of blood vessels in response to a rise in temperature. If rapid, automatic regulatory responses fail to reduce prediction error (PE), a prior is updated, forming a posterior probability that updates a lower sensory layer in the map hierarchy to ‘expect’ hotter temperatures. It is likely that updating of priors at lower layers of the map is therefore itself involuntary and automatic, and not subject to conscious deliberation or intentionality. On the other hand, at higher levels of the simulation map, conscious deliberation may select among several regulatory alternatives for minimizing PE. In the hot weather example, one might choose to seek shelter from the heat, or appraise the heat as welcome and attempt to enjoy the warm air. Whether one selects to regulate sensation to fit the prior, or allows the prior to be updated, is an important regulatory distinction that will be discussed at length below. For now, the critical point is that the ability for sensations to motivate regulatory responses is driven by the magnitude of the PE, the deviation from the priors at a given layer of simulation. The involvement of higher order cognition in this resolution process is determined by how high an error signal processes up through layers of the simulation map before it is resolved, with larger discrepancies being more likely to make their way up through the layers to reach conscious awareness.

The comparison between sensed and expected states, reminiscent of Higgins’ self-discrepancy theory (Higgins, 1987), serves two major functions: (i) to orient us to surprising physiological changes that require immediate attention, and (ii) to allow comparison between current and potential future states as a cue to action. In the face of a mismatch between sensed and expected states, ***active inference*** promotes responses intended to shape the interoceptive milieu to match expected states. For example, given an unexpected headache, one may feel an urge to take a pain-killer, in order to restore one’s desired, habitual pain-free state. Not all active inference requires overt behavioral intervention, or even awareness of a PE: some forms of active inference are physiological, wherein the autonomic nervous system regulates physiologically largely automatically and unconsciously to return the body to its expected state (Gu and Fitzgerald, 2014). On the other hand, when exposed to stressors that exceed one’s autonomic regulatory capacity, cascading PEs may reach levels of the simulation map that are accessible to conscious awareness. Such awareness prompts overt active inference, by which one interacts with the environment to approach an expected state (Seth, 2013). Critical to our definition, both physiological and overt forms of active inference seek to reduce PE by changing sensation to approach prior expectations rather than updating the priors themselves.

However, active inference is not the only way to reduce the disparity between current and desired states. One may also adjust one’s expected state to match the internal milieu. The process of updating the expected simulation map to more accurately reflect immediate sensation is known as ***perceptual inference*** (Seth, 2013). A person’s goals and attitudes toward interoceptive sensation may powerfully influence the form of inference made (**Box 2**).

**Box 2. How motivation shapes interoceptive inference.**

**FIGURE 1:**
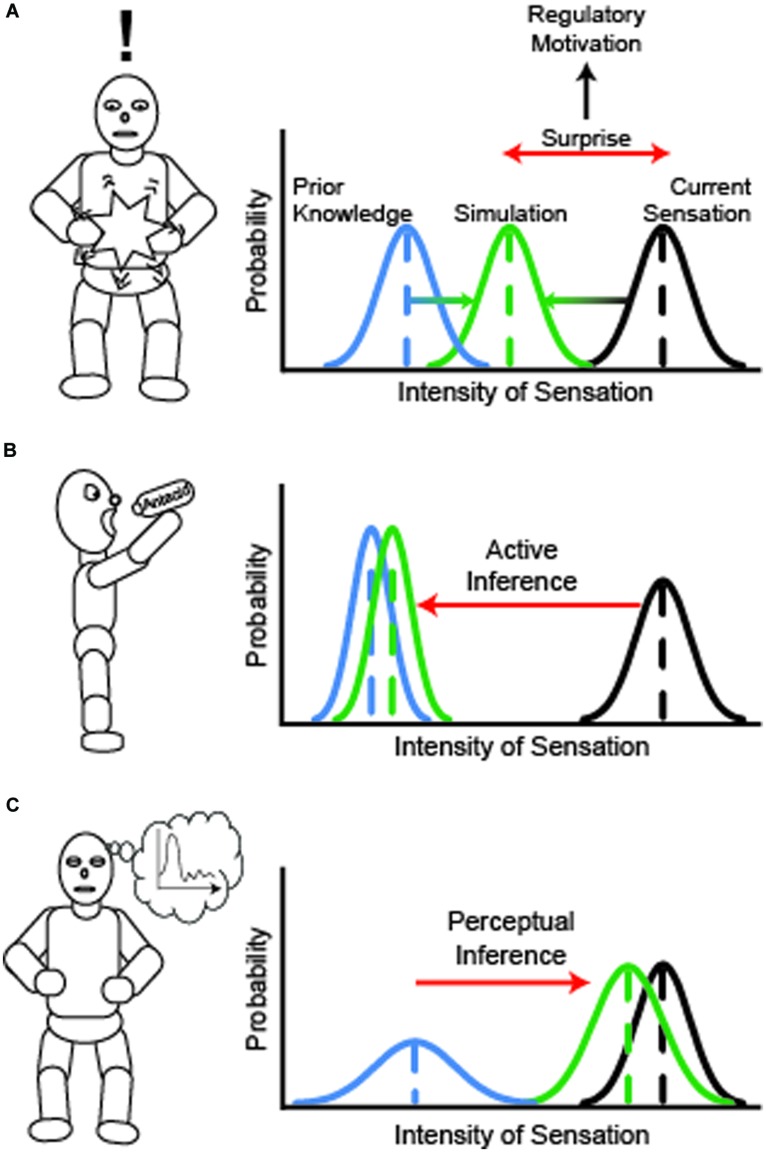
**A prediction error (PE) model of interoceptive inference, adapted from (Seth, 2013), in which interoceptive knowledge is represented by three terms: priors (blue lines), i.e., the probable body state as informed by prior events; sensation (black lines), the current sensory input from the body; and simulation (green lines), the current predicted body state based upon integration of current bodily feedback and prior learned contextual influences.** Critically, our model suggests that simulation rather than raw sensation is the closest construct to interoceptive awareness. **(A)** Unexpected interoceptive events, such as a stomach ache, are represented as a PE signal that motivates a regulatory response to minimize the error signal. The simulation distribution is displayed as equidistant from the sensation and prior distributions to indicate the potential for updating from both of these information sources. **(B)** Active inference reduces PE by weighting priors over current sensation. A high-specificity range of expected body states leads to large PEs from unexpected sensations, motivating attempts to modify internal states indirectly through cognition or behavior. Successful inference reduces PE by aligning incoming sensation to match the prior. **(C)** Perceptual inference reduces simulation error by weighting current sensation over priors, updating the simulation to fit sensation. A low-specificity range of expected body states lessens immediate PEs from unexpected sensations, lending interoceptive simulations (i.e., future priors) greater sensory accuracy. Successful perceptual inference reduces PE by updating the prior to match incoming sensation. Active and perceptual inference may co-occur dynamically over time, shifting attention between sensory updating and regulatory response.

Given the power of interoceptive signals to alternately capture attention and provoke regulatory behavior, achieving an optimal balance between active and perceptual inference seems central to successful self-regulation. The tension between interrogating perception and formulating a response has been addressed in recent models of interoception processing (Hankin, 2012; Paulus et al., 2012; Seth, 2013), and we elaborate on it here to discuss the role of contemplative practice in addressing the relatively underspecified process of perceptual inference. At the level of perception, interoception requires a mental representation of body state as well as tactile or other related sensory information within the simulation map, which is constrained both by bottom-up sensory inputs, and by top–down expectations based on the stored knowledge of prior interoceptive states and knowledge of one’s current context. Neither sensory afferents nor prior representations are necessarily accessible to conscious awareness, although particular layers of the simulation map may become the objects of awareness. Motivation within the model is generated as a response to discrepancies between the simulated and incoming interoceptive signal, which is also referred to as the prediction error or ‘surprise’ signal (**Figure 1A**). Incoming sensory signals are contrasted against prior information at a given level of the simulation layer. Discrepancies between the sensation and prior feed up through simulation layers, and generally trigger regulatory activities to maintain a homeostatic state. Ultimately, prediction errors are resolved by attributing causes to the unexpected signal, resulting in the creation of a posterior probability that cascades down simulation layers to update priors and reduce prediction error. The posterior probability determines the meaning of the signal and consequently the nature of the regulatory response.

Interoceptive *sensitivity* to unexpected, afferent interoceptive signals describes how readily a prediction error propagates up through simulation layers, serving as a basis for motivation in dynamic interoceptive representation. However, sensitivity alone does not speak to differences in how a person responds to the motivating discrepancy signal. Top–down cognitive factors in the forms of goals, causal appraisals, and contextual cues help to shape the motivated response. The tension between active and perceptual inference is often resolved by how one responds to the surprising mismatch between simulation and sensation. If current goals lead one to value *regulation* over *accuracy* of the interoceptive signal, priors at lower layers will be updated with an inferred explanatory cause (the posterior probability) from the discrepant layer that suggests overt active inference to address this cause. In this situation, surprise is minimized by down-weighting discrepant sensory information in favor of acting to restore a previously expected state (**Figure 1B**). In active inference, the simulation is closely aligned with the prior, and the individual attempts to shape incoming sensation to match the prior, thereby reducing prediction error. Conversely, if a person values accuracy over regulation, he or she will place high weight on sensory relative to prior information, updating the posterior to create a simulation that matches the unexpected afferent sensory information without attempting to restore a prior state (**Figure 1C**). In perceptual inference, the updated simulation departs from the prior and aligns with incoming sensory information; the posterior probability updates the prior rather than prompting efforts to change the sensory input, thereby reducing prediction error. In either situation, the discrepancy is minimized; the concept of sensory precision weighting allows the model to predict how such minimization occurs.

It is unknown how the different forms of inference will affect the distribution of priors in calculating prediction error. If perceptual inference allows for greater updating of priors, then it will likely promote greater variability of priors than active inference, which attempts to constrain the simulation to fit an existing set of priors. For perceptual inference, the distribution of sensory information is rendered more precise instead of the priors. These precise sensory distributions will have greater impact on higher level posterior probabilities, which feed back upon the simulation, making it more accurate while being agnostic to its effects on the prior distribution. If the body shows greater variation in sensation than in prior experience, then the priors will become less precise; if, however, it turns out that the body is displaying a sensory input that is more consistent than in prior experience, then one should expect to see more precise priors following updating. It is our yet untested hypothesis that for most people there is greater variation in the body than we tend to expect, and so the effect of contemplative practice will be to expand the range of anticipated experiences rather than hone in on any one experience, leading to a broadening of the prior distribution. The consequence of such broadening is that the simulation map will more accurately reflect sensory experience as it is less influenced by a rigid prior and more so by incoming sensation.

While active and perceptual inference both seek to minimize the disparity between sensed and expected states, they differ in their means of reducing this disparity. Overt active inference is a process by which an organisms acts to confirm/disconfirm attributed causes of unexpected interoceptive sensation, whereas perceptual inference acts to reduce the surprising nature of the sensation by broadening sensory expectations, reducing their inferential weight on the simulation layer. In many ways, this distinction is analogous to the difference between modern psychological and contemplative accounts of emotion regulation. While modern psychological models often discuss suppression, distraction, or reappraisal to alter the characteristics of the interoceptive signal (Gross, 2002), contemplative traditions make use of terms such as acceptance and equanimity, or simply continuous non-interfering observation, as ways of changing ones attitude toward sensation rather than attempting to change sensation itself (Mikulas, 2011). More recent secular adaptations of contemplative traditions almost universally feature integration of these perceptual inference strategies.

We note that this conceptualization of acceptance or equanimity as a ‘bottom–up’ perceptual rather than ‘top–down’ cognitive strategy may be controversial, but believe that this distinction is critical for understanding contemplative therapies’ intended mechanisms of action. Indeed, contemplative therapies may be valuable precisely because they challenge existing models of ‘top–down’ emotion regulation by introducing the idea that sometimes attempting to control or regulate emotional experience is itself the problem. In a system where perception and appraisal are seemingly obligatory, iterating steps in human experience, perceptual inference may facilitate emotion regulation by reducing overlearned and seemingly obligatory perception–appraisal associations. Appraisals will still follow perceptions, but they need not be so stereotyped and rigidly constrained.

The distinction between active and perceptual inference matters in everyday life: for example, consider a person experiencing a sense of restlessness, who infers that her unexpected restlessness stems from hunger. In this case, active inference rapidly pairs an unexpected interoceptive signal with an externally directed behavioral response intended to restore the interoceptive milieu to its expected, homeostatic state. In doing so, active inference shifts attention away from interoception itself, at least temporarily. If it turns out that the restlessness stems from some other source, such as workplace stress, active inference’s promotion of a shift from interoception to regulation reduces the opportunity to explore unfolding interoceptive signals. By contrast, perceptual inference involves reducing reliance on prior expectations in the face of an unexpected sensation. Through perceptual inference, the state of restlessness becomes the new expected state. From this perspective, the dynamic time course of arousal may be explored, including attention to conditions associated with changing interoceptive states. With time and reflection, perceptual inference might allow the individual to realize that her arousal was greatest when thinking about the workplace, and that the arousal was not actually a hunger signal. Over repeated applications of perceptual inference, prior expectations for arousal may change so that arousal following reflection on the workplace is no longer unexpected, but is instead a familiar consequence of such reflection. Knowledge of such conditioning may then afford new regulatory opportunities, a more adaptive set of priors for anticipating and explaining physiological arousal.

If perceptual inference provides grounds for personal insight, moving directly from an unexpected sensation to regulation (overt active inference) may reduce the opportunity for such insight. It is thought that such ‘knee-jerk’ regulatory responses to emotional arousal may be a factor that drives emotional eating behavior (Ouwens et al., 2009). In such cases, active inference does not lead to improved interoceptive accuracy, but instead maintains an error state. Indeed, overt active inference may require a masking or abstraction of nuanced and rich interoceptive signals for the purpose of promoting a rapid behavioral response.

It is not the authors’ goal to denigrate the importance of active inference in allowing human beings to flexibly and dynamically adapt to the world in which they are inextricably embodied. Many unexpected sensations do require ‘doing,’ an active regulatory response, rather than simply ‘being’ with unexpected sensation (Williams, 2008). Indeed the most adaptive behavior may come from iterative cycling between perceptual and active inferences. However, the use of active inference should come with an oft-unappreciated caveat: it seems inevitable that sensory granularity will be lost when one redirects attention away from the body toward response formulation. Sensory granularity here is understood as the ability to notice specific details of internal sensory experience such as subtle changes in sensation. We posit that granularity requires the capacity for sustained attention to sensation, or least the ability to flexibly shift back and forth between sensory monitoring and conceptual inference.

Degradation of sensory granularity is an important tradeoff in shifting to a problem-solving, active inference mode of processing. In many cases, the ability to notice changes in hunger and satiety levels as one eats is adaptive, empowering feelings of satiety to terminate the eating response and maintain homeostatic energy balance. Conversely, in our example above, realization that a sense of restlessness was not alleviated by food consumption is likewise important for ruling out eating as an appropriate regulatory strategy. A failure to return to interoceptive sensation following active inference can be seen as a fixation with a mode of ‘doing’ rather than ‘being.’ Such fixation may promote continuation of food intake and potentially disrupt homeostatic energy balances if hedonic eating drives are chronic (Lowe and Butryn, 2007). If one is able to respond to unexpected sensation through perceptual rather than overt active inference, the motivational importance of even powerful interoceptive signals may be mitigated, allowing for deeper inquiry and reflection into the conditioning underlying the interoceptive signal. In modern secular culture, it has been argued that the balance between ‘being’ and ‘doing’ has been skewed toward the ‘doing’ mode; it may therefore take work to rebalance regulatory dynamics to optimize one’s regulatory potential (Williams, 2008). Affording a transition from ‘doing’ to ‘being is consequently a primary aim of interventions such a Mindfulness-Based Cognitive Therapy (Segal et al., 2012).

In summary, the integration of active and perceptual inference in the predictive coding model allows for the shared influence of secular and contemplative traditions in accounting for how body sensation are regulated. The idea of overt active inference fits well with the existing scientific literature- the idea that we are the curators of our bodies, needing to regulate interoceptive perturbation. Active inference can serve a variety of ends, be they allostatic, aiming to restore the body to its homeostatic baseline (Craig, 2009; Gu and Fitzgerald, 2014), hedonic, aiming to achieve some desired pleasurable, energetic or tranquil state (Kringelbach, 2005; Paulus et al., 2009; Naqvi and Bechara, 2010), or even nihilistic, aiming for freedom from sensation altogether, as is found in placebo-based analgesia (Büchel et al., 2014). Perceptual inference, by contrast is often less specified in discussions of adaptive self-regulation.

A greater understanding of the mechanisms underlying perceptual inference is important, because high sensory precision weighting may be one avenue by which to effect change in habitual regulatory behavior. Indeed, high levels of sensory granularity may be critical in challenging interpretive biases during the translation of concrete, granular levels of the simulation map into more gross or abstract levels of representation. Through attention to unelaborated emotional or physical sensation, a person may discover significant levels of tension and/or psychological distress associated with the body that were not previously in awareness, revealing a need for greater self-care (Price, 2005; Price et al., 2012a, 2013). Conversely, interoceptive attention may reveal that an allostatic habit such as self-medication is not always necessary in the face of noxious interoceptive sensation (Kabat-Zinn et al., 1985). Recent research suggests that the brains of experienced meditators demonstrate reduced PEs to passively viewed reward (Kirk and Montague, 2015), indicative of less clinging or attachment to positive outcomes that may serve as a basis for addictive behavior. Constructively, perceptual inference may also make people aware of inner resources, affording access to previously hidden capacities for calm, contentment, and appraisals that things are going well just as they are. In this way, every moment requires a choice between perceptual and active inference. And yet, how does a person know which information to use or whether he or she has enough information to act adaptively in response to their goals? When should one switch from perceptual to active inference? The ability to respond appropriately to interoceptive perturbation is a challenging problem that can serve to determine a person’s sense of well-being across the lifespan.

With respect to the relevance of contemplative practice, a final point warrants mention surrounding the accessibility of active and perceptual inference. While ideally we are presented with a choice between the two error-minimizing responses, often this is not the case. While active inference appears to be a ubiquitous, evolutionarily conserved regulatory strategy (McBride, 2012), reframing unexpected sensations as a perceptual problem may require the cultivation of attentional capacities, intentions, and attitudes that are less intuitive. Our bodies perform physiological active inference even before we are born (Moor, 1968), and learning to apply overt active inference in the form of clothing, feeding, and otherwise caring for ourselves is a complex but near-universal feature of childhood development. Perceptual inference at these early stages may not be possible given limited cognitive resources or maturity of perspective, and indeed it may be counter-productive to an individual’s mastery of overt active inference responses. As we mature, however, we realize that not all unexpected sensations can be corrected, or that our habitual, reflexive corrective habits are actually maladaptive. While behavioral therapies address this problem by working to bring such overt active inference habits to awareness so that they may be fruitfully restructured, perceptual inference may require development of a qualitatively different skill set. Thus it is important to consider that active and perceptual inferences are not equally available, and that perceptual inference tends to be the less available of the two without specific types of learning experiences.

## Interoception in Health, Disease, and Well-Being

It has been proposed that mental representations of selfhood are based fundamentally on embodied sensory experience, supporting a sense of the self in the world that is crucial for interacting with the environment (Seth, 2013). From this perspective, greater accuracy of interoceptive self-representation promotes greater moment-by-moment adaptation, whereas dissociation from accurate representation can lead to dysregulation. Accordingly, many contemporary health problems involve dysregulated interoceptive processes, including affective disorders (Paulus and Stein, 2010), addiction (Naqvi and Bechara, 2010), eating disorders (Garner et al., 1983; Pollatos et al., 2008; Herbert and Pollatos, 2014), chronic pain (Schmidt et al., 1989), dissociative disorders (Hankin, 2012; Michal et al., 2014; Sedeño et al., 2014), post-traumatic stress disorder (PTSD; Wald and Taylor, 2008), and somatoform disorders (Mirams et al., 2012; Schaefer et al., 2012). Understanding how interoceptive processes influence representations of the self in the world and self-regulation may lead to improved disease and treatment models (Pollatos et al., 2005).

### Presence and Agency

One approach to understanding dysfunctions in interoceptive awareness is to apply the predictive coding framework as an explanatory model distinguishing adaptive and maladaptive processing. Toward this end, two extensions of the model have been proposed, describing how inference processes outlined above operate to minimize PE (Seth et al., 2011). The first of these concepts is *presence*: presence is thought to arise when interoceptive and/or exteroceptive PE signals are successfully minimized. In Seth et al.’s (2011) account, such error minimization has been attributed to either overt or physiological active inference; as one successfully resolves unexpected sensation through autonomic or behavioral responses, a feeling of engagement and connection with one’s body and environment ensues. However, we propose that, from a contemplative perspective, presence is accessible through perceptual inference as well.

There are several ways that PEs may be reduced to give rise to presence: in a virtual reality environment, interoceptive signals may be successfully masked by salient external signals, such as seeing oneself walking in the absence of proprioceptive motion signals, leading to a sense of presence through acceptance of the external signal as one’s own embodied state. Given predictable and salient external cues in a virtual reality environment, a sense of presence may come more easily than trying to detect and match faint and chaotic interoceptive cues within the noisy internal milieu. By contrast, in contemplative practice the sense of presence through a variety of visceral experiences may broaden the distribution of interoceptive expectations (priors), reducing the precision of those priors, and thereby minimizing the potential for PE. In other words, in a simulation map that allows for great variation in sensory inputs, relatively few visceral sensations are extreme enough to create a PE demanding a regulatory response.

We suggest that with successful iterations of perceptual inference, the influence of prior expectations is thereby weakened, leading to feelings of automatic simulation map updating: effortless presence (Sjölie, 2014). While healthy individuals likely experience such effortless presence as a matter of course, it is likely that such experiences operate across a continuum of effort, with extreme difficulties in achieving presence manifesting as depersonalization and derealization disorders (Seth et al., 2011). However, even within the healthy population, the degree to which presence is experienced and maintained may be dependent upon regulatory strategies and habits. A person whose regulatory habit is overt active inference may spend a great deal of time pursuing idealized interoceptive states, rather than learning to allow autonomic regulation to more subtly achieve and maintain such states. Such a person would require greater effort to achieve feelings of presence. Furthermore, a reliance on active inference in general may reduce the possibility for presence when a sensation cannot be easily mapped to an active regulatory pathway, be it through overt behavior or autonomic regulation. In such situations, effortless presence may seem like an important but unachievable state, similar to the ideal of accepting one’s negative thoughts and feelings for individuals with a history of depression or anxiety (Pauley and McPherson, 2010). For such individuals, learning to reduce PE through perceptual rather than active inference may constitute a radical shift in self-processing, the introduction of a new regulation strategy based on acceptance rather than control. It is unknown how variable feelings of presence are in the general population; but we would hypothesize that effortless experiences of presence are fleeting at best in the frenetic, “what’s next”-paced endemic of modern Western culture.

As an example of presence functioning adaptively, one might imagine the perception and then acceptance of an unexpected, transient increase in heart rate. Following the initial upset, a person may engage in perceptual inference, exploring the interoceptive signal, which we operationalize as an attempt to update the simulation map to match this signal. Acceptance would then occur when perceptual inference successfully simulates the elevated heart rate as the normal, expected state. By accepting the internal change, the motivational force of the physiological change is minimized without requiring the suppression of either expectations or interoceptive signals. By contrast, the use of overt active inference to resolve unexpected events has been associated with the sense of *agency* (Seth et al., 2011), a feeling of control over one’s actions in the world, that one can act to produce particular results. Agency can be inspired both by direct regulation of bodily sensation, as well as through changes in behavioral patterns that indirectly contribute to one’s interoceptive state. In the context of our discussion of interoception and well-being, the sense of agency is important for supporting a sense of responsibility for self-regulation. This applies both to conventional forms of overt active inference, such as quitting a stressful job and going for a less paid but more livable employment, but also to contemplative practices such as learning that one can reduce feelings of anxious arousal through breath monitoring or other regulatory activities.

Presence and agency are important in that they establish norms for experience and control that guide behavior and ultimately determine one’s sense of well-being. In anxiety disorders, for instance, agency is achieved through withdrawal from stressful situations, a form of active inference that is effective in the short term but ultimately maladaptive, as that person never learns that the feared outcome is unlikely to be true. In anxiety, PEs are augmented- a person may experience a strongly aversive response to a previously neutral stimulus (Paulus and Stein, 2006). This aversive response is then augmented rather than extinguished when the anxious individual makes catastrophic appraisals of the aversive sensation that are paired with active inferences to manage these perceived catastrophes. For example, if a racing heartbeat during exercise is appraised as a heart attack; active inference requires a trip to the hospital. If rapid breathing prior to public speaking is appraised as lack of ability that will lead to embarrassment, active inference requires social withdrawal. Faced with threats to agency, maladaptive behaviors are reinforced, which ultimately interferes with exploration and modification of one’s relationship to stressful events. From a predictive coding model, chronic stress may lead to a reduction in the precision of PE coding in favor of strong priors around the recognition of aversive sensation and confirmatory active inference behaviors to alleviate that sensation. While it is unpleasant to experience unexpected aversive arousal, it is this secondary appraisal around agency, the threat to control over the situation that is the cause of deep distress. The idea that higher order constructs such as agency are at the heart of subjective well-being is a central theme of modern appraisal theory (Scherer et al., 2001), just as presence lies at the heart of contemplative accounts of well-being (Brown and Ryan, 2003). These theories serve to reinforce the point that subjective mental health stems from our inferences about coping capacity, for which each attempt at perceptual or active inference serves as a momentary test. If imprecise PEs prompt inefficient or maladaptive active inference behaviors, inferences of coping failure are likely, exacerbating feelings of powerless and inefficacy and contributing to the deleterious impact of the stressor.

In its ability to link higher order appraisals of self-worth to momentary regulatory acts, the predictive coding framework is helpful in understanding many other disorders, because it suggests that well-being requires active sustenance, i.e., that presence and agency must be continuously constructed through successful reduction of PE (Paulus, 2007), regardless of the balance between active or perceptual inference. This perspective stands in contrast to notions that isolation from our bodies and the world is preferential except to alert us about negative events. Instead, it is the lack of integration of accurate interoceptive simulations into higher order representations that is hypothesized to underlie dysregulated cognition and behavior. In this observation there is considerable convergence with contemplative perspectives. For example, following trauma, some individuals may experience dissociation from bodily experience, as is found in some forms of severe depression and PTSD (Feeny et al., 2000). In such cases, the priors associated with body error signals may have been linked to traumatic experiences, which are too powerful to be actively controlled (active inference), and too aversive to be accepted or, as in many contemplative orientations, altered simply by being observed (both instances of perceptual inference). Since the error signals cannot be minimized, the simulation map itself may be suppressed in the form of *experiential avoidance*, a form of protective habituation to powerful and uncontrollable interoceptive signals, or *behavioral avoidance* of provocative situations altogether. Such inhibition may amount to a reduction of the precision of priors in the map, so that only very powerful sensory signals are able to trigger an upward flow of PEs toward higher simulation layers. While avoidance provides momentary relief from the attentional pull of PE signals, inhibition of interoceptive awareness may also create its own host of psychosocial issues, both through the obfuscation of important interoceptive cues, the absence of desirable interoceptive sensations, and the maintenance of dysfunctional beliefs about the world and their relation to it (Paulus and Stein, 2010). Indeed, the severity of somatic dissociation has been linked to greater susceptibility to experimentally induced somatic illusions, suggesting an over-reliance on prior knowledge that interferes with current sensory input (McKenzie and Newport, 2015). Many contemplative traditions begin from the assumption that a suffering individual’s interoceptive integration may be dysfunctional or deficient; to rectify this situation, attention to the body may delay cognitive appraisal, creating space to restructure the integration process (Shapiro et al., 2008).

### Interoceptive Training in Clinical Practice

Specific examples of dysfunctional interoceptive integration are myriad in psychological disorders. For instance, when an individual with severe attachment disturbances considers close social interaction, there is often an immediate discrepancy between expected and actual feelings of connectedness, providing a new source of interoceptive surprise that may be anxiously appraised (Rector et al., 2006; Gilbert et al., 2012). Similarly, it is not uncommon for a person with a severe history of trauma to be so anxious that it is not possible to experience the positive sensations of close interactions (Fogel, 2009; Frewen et al., 2012). In the absence of new interoceptive information, such fears and concerns may take on the quality of rumination, perseverative thinking that is associated with affective disorder vulnerability (Nolen-Hoeksema, 2000). The predictive coding framework provides an explanation for how rumination can dominate attention: when simulation maps operate with low perceptual weighting, attention is more often drawn to active regulation, leaving little opportunity for experiences of interoceptive presence, a feeling that a sensation is acceptable and tolerable, that may challenge sustained bleak and dysphoric expectations. Contemplative training is largely oriented to increasing one’s capacity to de-habituate such coding. Commonly, such cognitive distortions of reality may continue unfettered, leading to a downward spiral that characterizes the chronic and recurrent nature of affective disorders (Raes et al., 2008).

And yet, despite the importance of perceptual inference for well-being, we should stress that such inference alone is not a panacea. Perceptual experience is not easily divorced from subsequent appraisal, and so even accurate and precise interoception may activate powerfully conditioned negative associations and appraisals (Treleaven, 2009). For example, in anorexia nervosa, patients may possess powerful associations between awareness of bodily states associated with starvation and a ‘doing’ mode that focuses on the control of eating, shape and weight, ultimately culminating in interoceptive suppression once more (Watts et al., 2007; Park et al., 2011). In anorexia, bringing awareness to the body will initially be met with an increase rather than decrease in maladaptive behavior. Thus there is a reason that interoception becomes disrupted, and skillful guidance is particularly important in restoring interoceptive access.

If a regulatory framework is not in place, such as the capacity for acceptance of ambiguous or challenging sensations, restoring interoceptive access- or advocating contemplative intervention- could introduce new trauma. For example, enhancing interoceptive awareness without compensatory regulation may be maladaptive to a severely depressed and suicidal individual or someone with acute pain from a severe burn. In many cases, however, it seems that specifically trained, skillful clinical or contemplative guidance can successfully address the challenges inherent to interoceptive re-engagement. For example, research with women in treatment for substance use disorder suggests that when they are encouraged to discover the connection between physiological and emotional distress, they often learn that they have the capacity and skills to attend and negotiate emotional stress. Enhanced interoceptive awareness provides nuanced cues for self-care that facilitate emotion regulation, reducing conditioned substance-use responses to stress, and allowing such patients to maintain sobriety (Bowen et al., 2007; Price et al., 2012b).

The need for a skillful interpretive framework is apparent in psychometric research on mindfulness of body sensation, such as the ‘observe’ facet of the five-factor mindfulness questionnaire (Baer et al., 2008). This research on the questionnaire suggests that high levels of this ‘observe’ facet are associated with mixed health outcomes in the general population, but with more uniformly positive outcomes in those who are relatively trauma-free and have received a background in contemplative training. A recently published report pointed out that the commonly assessed ‘observe’ facet, becoming aware of bodily changes, may change with these approaches to a much smaller degree than the regulatory aspects of interoceptive awareness, that is, how the body is used for self-regulation in daily life (Bornemann et al., 2014). In this case, a multidimensional self-report measure was sufficient for distinguishing between aspects of interoceptive change, and serves as an important precedent for further clinical research. It should be noted, however, that the bulk of the clinical research discussed in the following section has employed only more global qualitative reports of interoceptive change, a finding which may in fact be driven by training effects on only a subset of interoception’s many facets.

### Clinical Examples in Patients with PTSD or Chronic Pain

To clarify the relevance of interoception in the promotion of well-being, it may be useful to consider examples where the cultivation of adaptive interoception has yielded clinical success. Emerging research suggests that contemplative practices may powerfully support the process of interoceptive re-discovery. In the treatment of women with a history of interpersonal sexual violence, participants learn to engage in perceptual inference, recognizing when sensory cues of dissociation are triggered emotionally, allowing them to maintain awareness of their bodies instead of dissociating from those sensations into habitual, active regulatory responses. Over time, participants discover that their bodies can be a helpful informative resource rather than a source of threat signals that should be avoided. Thus, body sensations not previously incorporated into awareness can be allowed into phenomenological experience, and can be more appropriately integrated into self-schemas. These experiences can lead to a greater sense of safety in the world, greater ability to engage in intimate interactions with spouse/partner without dissociating, greater ability to negotiate stressful environments and interactions, and lastly a greater sense of wholeness and empowerment (Price, 2005, 2007). It remains to be determined whether these contemplative practices increase interoceptive accuracy directly, or instead support other elements of our interoceptive taxonomy, such as increasing sensitivity to interoceptive events and moderating subsequent regulatory habits.

A further example of contemplative practices’ potential lies in the psychological management of chronic pain, in which the most commonly applied approach is currently a combination of cognitive reframing by cognitive behavioral therapy and attentional distraction (Hoffman et al., 2007). Recently, however, it has been questioned whether distraction works for chronic pain as well as it does for acute experimental pain (Goubert et al., 2004). One reason for reconsidering distraction techniques is that part of chronic pain pathology may actually revolve around maladaptive fear associations that impoverish interoceptive processing and exacerbate pain syndromes, conditioned withdrawal associations which distraction would only serve to reinforce rather than challenge (Zaman et al., 2015). Instead, “interoceptive exposure” may be an alternative (Craske et al., 1997; Boswell et al., 2013). Yoga includes interoceptive training, is associated with decreased prefrontal brain activity and has shown benefits for pain management (Villemure et al., 2013). Keeping in mind concerns that interoceptive awareness is not always beneficial, helpful, or tolerable, it would seem that these and other examples suggest that in many cases, careful guidance of attention, informed by contemplative theory, and with case-sensitive guidance, may allow people to reconnect with their bodies with great therapeutic potential. With appropriate support, patients may learn to tolerate negatively conditioned experiences of the body, eventually accepting their prepotent aversive response, which frees cognitive resources for reconstruction of the appraisal process (Park et al., 2012).

In summary, interoception is important for well-being, at a pragmatic level of maintaining desired physiological states within the body, but also at an epistemic level for its contributions to perceptions of presence and agency. Indeed, many psychological disorders are characterized by disruptions to presence and agency in the form of dissociation or hopelessness, or by maladaptive solutions to agency violations, as is found in anxious withdrawal. Contemplative training may be effective to restore adaptive interoceptive dynamics that address these violations, keeping in mind the caveat that the reframing of interoceptive signals must be carried out with care and support to increase tolerance for aversively conditioned sensation. How such practices may accomplish this restorative feat is the topic of the next section.

## Contemplative Practices for Revitalizing Interoception: The Example of Mindfulness

Given compelling evidence that interoceptive processes are integral to many forms of affective and related disorders, we might ask how interoception can be restored to an optimal state. Clinical experience suggests that trusting in body signals as potentially decision-guiding information and valuing the body as an important resource in directing one’s behavior may be key conditional precursors for this change (Mehling et al., 2011). In the predictive coding framework, trust or acceptance of interoceptive signals fits with our discussion of increased sensory weighting, perceptual inference, and the cultivation of feelings of presence. While modern scientific traditions have much to say about the mechanics of the body, how such mechanics can be used to cultivate a more embodied phenomenological state has not historically been a focus of these traditions. It is in this effort to understand the cultivation and sustenance of presence and agency that contemplative science may be of value.

Many body-based contemplative practices involve explicit direction of attention to interoceptive sensations. The exploration of interoceptive awareness under many other names is central to Asian contemplative, medical, and philosophical traditions. Importantly, these are presented in a philosophical context of exploring errors about the self and individual subjectivity that result in part from ignoring, misinterpreting, or missing more subtle levels of an individual’s interoceptive experience. Such contemplative practices, although they may have numerous other goals in addition to training interoceptive awareness, offer a method for training in such awareness and, in many cases, for reorienting experience from, for example, distraction to attentional control, effort to ease, separateness to connection. All these shifts are deemed beneficial. In this section, we will focus on mindfulness meditation traditions as they contribute to a sense of presence through largely stationary interoceptive attention practices. It is however important to note that other, movement-based traditions such as yoga or tai chi may be especially well-suited toward the cultivation of agency. A great deal of research is needed to explore the effects of particular practices on the predictive coding of bodily awareness.

Among contemplative traditions, mindfulness has recently received a particularly strong representation in modern sciences. There is an extensive literature on the health benefits of mindfulness approaches- for overviews see (Grossman et al., 2004; Chiesa and Serretti, 2009; Hofmann et al., 2010), particularly in the area of preventing relapse in those vulnerable to depression (Goyal et al., 2014). It should be noted that interoceptive training is only one aspect of mindfulness interventions, which also emphasize changing one’s relationship to thought content. Nonetheless, such interventions may represent the most prominent introduction of interoceptive training in the West. Mindfulness approaches may be particularly efficacious in reducing somatic dissociation from chronic pain and sexual trauma (Price, 2007) but also in other mental health disorders such as substance use (Price et al., 2012b) depression (Williams et al., 2013), anxiety (Hoge et al., 2013) and eating dysregulation (Daubenmier et al., 2011). There are suggestions that interoceptive mindfulness practices yield changes across a variety of cognitive domains, including self-reference (Brown and Ryan, 2003; Farb et al., 2007; Ingram et al., 2011), attention (Jha et al., 2007; De Raedt and Koster, 2010), emotion regulation (Ortner et al., 2007; Jha et al., 2010), pain perception (Zeidan et al., 2010a; Gard et al., 2012), agency (Allen et al., 2009), and feelings of social connectedness (Hutcherson et al., 2008; Neff and Germer, 2013); see (Hölzel et al., 2011) for an integrative review.

Modern scientific definitions conceptualize mindfulness as an open, engaged, and non-judgmental awareness of the ongoing flux of present moment experience, including internal experiences of sensations, thoughts, and feelings, as well as exteroceptive sensations. It has been argued that one of the primary and early means by which mindfulness benefits its practitioners is by anchoring attention to interoceptive signals such as the breath or body sensation (Mehling et al., 2011; Kerr et al., 2013; Farb et al., 2014), an idea supported by training-related reports of both increased subjective body awareness (Mehling, 2001) and increased strength of brain networks dedicated to interoceptive processing (Farb et al., 2013). *Focal attention* to bodily sensations is at least one method by which mindfulness training shapes cognition (Hölzel et al., 2011); with stability of interoceptive anchoring, practitioners also engage in *open monitoring* of experience, which, from the modern secular perspective of using contemplative practice for therapeutic effects, reveals and weakens maladaptive reactive patterns so that they may be adaptively modified (Lutz et al., 2008; van den Hurk et al., 2010). From a predictive coding framework, body-focused contemplative practices may alter interoceptive processing by shifting regulatory habits from active to perceptual inference, increasing bottom–up integration of what is happening in the body rather than attempting to alter body sensation to fit top–down expectations of what should happen in the body (Pagnoni and Porro, 2014). Since even low level perceptions of interoceptive sensations commonly rely on the integration of both sensory and expectation processes, engaging in a contemplative interoceptive practice may reduce the precision of prior expectations, reducing the ability of priors to trigger PEs in response to incoming sensations. The consequence of this widening of acceptable sensory inputs may therefore allow for more accurate and dynamic representations of sensation, yielding more nuanced, and adaptive behavioral responses.

The wealth of empirical studies on mindfulness training mechanisms makes it a useful starting point in discussing how contemplative practices may impact interoceptive processing. Below, we describe several domains in which contemplative traditions such as mindfulness may positively impact interoceptive processing, acknowledging that these domains likely interact and support each other in an iterative and dynamic (rather than linear) manner.

### Enhanced Sensitivity

Contrary to the perceptions of even experienced meditators, mindfulness does not appear to generally increase interoceptive sensitivity when assessed in laboratory settings, at least where the most popular metrics of interoceptive accuracy, heartbeat perception, is concerned (Khalsa et al., 2008; Parkin et al., 2013). Mindfulness may, however, increase interoceptive sensitivity in domains that are the foci of meditative practice, such as sensation of the breath (Daubenmier et al., 2013), or interoceptive cues indicating the presence of subtle reactive patterns. Even brief body-scan meditations reduce errors in a subtle somatic signal detection task (Mirams et al., 2013), similar to the enhanced tactile acuity related to movement-based tai chi practices (Kerr et al., 2008). Furthermore, mindfulness training appears to alter interoceptive attention tendencies, focusing attention on interoceptive sensations rather than cognitive appraisals of such sensations (Garland et al., 2012), in keeping with a model in which contemplative practices shift regulatory strategy from active to perceptual inference. As such, sensitivity enhancements following mindfulness training may constitute a kind of ‘embodied ethic’ that promotes interoceptive attention deployment, a daily commitment to perceptual inference rather than an enhancement of sensitivity itself (Grossman, 2015).

One consequence of increased perceptual inference is increased *granularity* of interoceptive experience, reducing the self-appraised emotional impact of experience in favor of enhanced clarity and sensitivity to subtle emotion provocation (Nielsen and Kaszniak, 2006). Such sensitivity may manifest at multiple levels of the simulation map, allowing practitioners to recognize subtle, temporally extended dynamics of physiological arousal, dynamics that would ordinarily be obfuscated by definitive cognitive appraisals in response to early perturbations. The benefit of enhanced granularity is an opportunity to learn more about one’s body and its conditioning in the world. Indeed, mindfulness practitioners show increased accuracy between subjective and objective measures of body sensitivity, an index of the relative sensitivity to different body regions (Fox et al., 2012), and increased coherence between physiological and subjective states (Sze et al., 2010). Such coherence allows for greater appreciation of how stressful situations impact both mind and body, increasing the chances of adaptive regulatory action. Furthermore, because interoception engages the same neural pathways supporting pro-social emotions such as empathy (Singer et al., 2009), increased interoceptive sensitivity may also lead to improved social function, although direct evidence for this relationship is still needed.

### Enhanced Non-Reactivity

The deeper benefits of contemplative practices lie in leveraging non-reactivity to generate adaptive regulatory insights. The predictive-coding model can account for the availability of such insights through the idea of *precision weighting*, i.e., how much a person divides finite metabolic and attentional resources between (i) representing, exploring, and accepting unexpected sensory signals, i.e., PEs, and (ii) maintaining prior expectations. As discussed, attention can lead to greater precision weighting of sensation over priors, which in turn promotes perceptual as opposed to overt active inference (**Figure 1**). Both perceptual and overt active inferences require the use of metabolic resources, as evidenced by the feelings of effort when one first engages in either process. However, only overt active inference engages cognitive elaboration, requiring cognitive appraisal of the many action affordances available in response to interoceptive perturbations. Given that overt active inference is likely to consume considerably greater metabolic resources, individuals must attempt to optimize the precision of incoming sensory data and priors to minimize wasted regulatory effort (Fotopoulou, 2013). Unfortunately, for individuals with strongly conditioned regulatory tendencies, i.e., highly precise tuning of priors, this minimization may prove difficult in the face of seemingly automatic and obligatory regulatory responses. Resolving the disconnection between the ideal of regulatory efficiency and the reality of conditioned active inference is a non-trivial problem.

We hypothesize that one major benefit of contemplative interoceptive practice is that it provides a pathway toward perceptual inference in the face of such conditioning, effectively relaxing appraisal tendencies. While still demanding attention, perceptual inference frees higher order cognition to arise and pass without particular attachment to a given appraisal of the simulated state. In other words, perceptual inference frees higher order resources to allow metacognition, representation of the mind’s current state. The simple act of observation itself, as mentioned, can create a shift, possibly even without setting in motion higher-order resources. The idea that one’s thoughts can be viewed as transient mental events rather than as cues to immediate action is often referred to as *decentering* or *reperceiving* in the mindfulness literature (Shapiro et al., 2006; Fresco et al., 2007), and may be an adaptive consequence of responding to arousing experiences using perceptual rather than active inference. Decentering may also be related to the intentional arc, which sets mindfulness practice in motion and sustains it. Mindfulness training seems particularly well-suited for promoting decentering relative to progressive muscle relaxation or loving kindness interventions (Feldman et al., 2010), perhaps due to the explicit enhancement of sensory precision weighting at the expense of active inference. Such decentering may not be an end unto itself, but also afford flexible ‘re-centering’ on habitually ignored sensations that constitute meaningful, constructive, and positive experiences. Accordingly, mindfulness experience has been associated with increased cognitive flexibility (Moore and Malinowski, 2009). In this way, dedicated perceptual processing of interoceptive signals may serve to enhance conceptual non-reactivity to experience that in turn promotes well-being.

### Enhanced Regulation

In the metacognitive space that is afforded by enhanced non-reactivity, multiple interoceptive appraisals may be observed as arising and passing. From a decentered perspective, a person may become aware both of her most prepotent, i.e., frequently and powerfully occurring, appraisal tendencies, but also of alternative appraisal options. From awareness of multiple options, flexibility of choice can be directly experienced, thereby allowing for novel, creative, and potentially more adaptive appraisals and actions over time. As an example, a person in a stressful public speaking situation may use mindful attention to notice her elevated physiological response. From this perspective, appraisals of threat and incompetence may rapidly arise, but with continued attention toward sensory perception, such appraisals may also pass, interspersed with weaker, and less frequent appraisals of potential failure, support from loved ones, and feelings of determination. With this greater tableau of appraisals before her, the speaker may elect to appraise her arousal as a surmountable challenge rather than an imminent threat that requires withdrawal from the situation. It is through this process of forming insight that interoceptive attention may iteratively shift engagement away from habitual appraisals to promote experiences of insight and choice.

With practice, iterations of weighting attention toward perceptual inference may allow an individual to more permanently decondition and eventually replace appraisals of environmental stressors as threat with exploration of such stressors, appraising them instead as challenges to be explored, a distinction with important consequences for reducing physiological stress (Tomaka et al., 1993). Over time, this process of overcoming habitual dysphoric or catastrophic appraisals in favor of a less rigidly deterministic simulation map may result in positive trajectories for transformation, upward spirals in the promotion of well-being (Garland et al., 2010, 2011).

One example of adaptive sensory precision weighting in mindfulness can be found in a study of pain perception. Pain perception is theorized to have both a sensory component, representing the intensity and location of the interoceptive signal, and an affective component, representing the signal’s motivational relevance in term of pleasantness or unpleasantness (Melzack, 1975), a distinction supported by neuroimaging research (Tölle et al., 1999). In one recent study (Farb et al., 2013), mindful attention in experienced meditators was associated with increased attention to bottom-up signals as reflected in increased posterior insula activation, a region of primary interoceptive representation (Craig, 2002; Farb et al., 2012b), but decreased top–down processing as reflected in decreased lateral prefrontal cortical activity, a region involved in cognitive appraisal (Ochsner et al., 2004). These neural changes were linked to a change in the pain experience, such that pain was perceived as similarly intense but less unpleasant during a state of mindful attention. In the absence of cognitive judgment, cognition may be freed to consider alternative interpretations of sensory states. From a regulatory perspective, such freedom allows an individual to explore different forms of active inference, and may perhaps even reveal that active regulation of interoceptive signals is no longer necessary.

### Enhanced Insight

While perceptual inference, by definition, precludes immediate cognitive elaboration on sensation, in the long term, a greater corpus of interoceptive information provides a richer set of data from which to investigate habitual sources of interoceptive perturbation, to identify the relationship between inner somatic experience and cognitive experience, and one’s internal responses to outside events and stimuli. This meta-cognitive awareness may lead to *insight,* recognition of how events, emotions, thoughts, and bodily sensations relate to each other (Lavie et al., 2003; Sze et al., 2010). For example, recent studies in body ownership show that a sense of one’s body can be manipulated by experimentally induced visual and tactile feedback (Ainley et al., 2013; Suzuki et al., 2013) but those with greater interoceptive accuracy (as assessed by heartbeat detection) are less susceptible to illusions of body-ownership (Tsakiris et al., 2011). This ‘rubber hand illusion’ is a good example of prioritizing attention toward priors for the interpretation of visual input over attention toward PEs stemming from afferent interoceptive signals from the hand. For insight to occur one need not have perfect accuracy in such interoceptive signal representation; however, insight could emerge as a process of weighting sensory PEs over priors, leading to a higher-fidelity simulation of body state. In theory, contemplative practices could similarly reduce false inferences about the relationship between one’s body and the world.

As interoceptive signals inform emotional experience, contemplative practice may promote a cycle of awareness of the contingencies between environmental triggers, bodily responses, cognitive appraisals, and emotional experiences, knowledge which can then be leveraged to regulate cognition and behavior in the service of emotional well-being. We argue that this process optimally occurs when interoception is viewed as foundational to emotional experience, and thus interoceptive attention becomes a basis for engaging in emotional processing, enhancing awareness, and regulation of rapidly escalating emotional responses to stress. For example, in a study for women in substance use disorder treatment, those taught interoceptive awareness and related skills for self-care perceived such awareness as facilitating their ability to identify, accept, and process their emotions, key regulating factors that they attributed to successfully preventing relapse to substance use (Price et al., 2012a). Through these introspective cycles, insight is fostered that radically modifies inference of interoceptive signals, toward perceptual over active inference, exploring causal factors rather than requiring an immediate inference and response to interoceptive causality. Despite such enticing claims, more research is needed to substantiate the idea that mindfulness practice helps to alter the extent to which particular interoceptive signals are attended to and received with finer granularity in daily life.

### Enhanced Presence and Agency

If the link between perceptual inference and insight can be empirically validated, there are many downstream benefits that may ensue. For example, mindfulness’ ability to curtail habitual reactive tendencies may enhance one’s sense of *presence* and *agency*, and indeed mindfulness training has been associated with increased motor control during perceptual-motor conflicts (Teper and Inzlicht, 2013). Enhanced perceptual-motor integration reflecting increased agency may impact self-representations related to one’s ability to control the environment, which may have important consequences for one’s sense of well-being. Minimally, the increased weighting of interoceptive signal may increase interoceptive accuracy, decreasing the impact of dysfunctional simulations and self-representations on cognitive and behavior, as previously proposed (Farb et al., 2012a). For example, depression is characterized by external locus of control, learned helplessness, and low self-efficacy, and some evidence suggests depressed people have poorer interoceptive accuracy (Ehlers and Breuer, 1992; Pessoa and Ungerleider, 2004; Dunn et al., 2007; Pollatos et al., 2009), whereas mindfulness training appears to bolster self-efficacy (Chang et al., 2004). Furthermore, mindfulness training has been associated with normalization of the gait pattern of depressed individuals, suggesting concurrent changes in proprioceptive and emotional responses (Michalak et al., 2011). Presence and agency appears to matter for physiological self-regulation: the rubber hand illusion, in which visual-tactile illusions decrease a sense of ownership over one’s arm, results in decreased skin perfusion and temperature (Moseley et al., 2008), and increased stress hormone release in that arm (Barnsley et al., 2011), without providing commensurate pain relief in that arm (Mohan et al., 2012). These findings suggest that a strategy for minimizing interoceptive PEs through substitution of interoceptive inputs with exteroceptive inputs leads to dysfunctional, and, in the long run, unhealthy physiological changes in the respective bodily region. Conversely, we would hypothesize that minimization of interoceptive PE by increased precision weighting of sensation over priors may create a sense of embodied presence and be able to reverse such dysfunctional physiological changes.

Over time, an increased sense of presence and agency may also begin to enhance higher order self-representations such as self-esteem. Through this process, a person may hold greater confidence in his engagement with interoceptive inference, which may in turn reinforce the exploratory cycling between perceptual and active inference, again promoting upward spirals in the promotion of well-being (Garland et al., 2010, 2011). It remains to be tested whether enhanced interoceptive attention may produce differential cognitive-emotional-physiological effects when placed in different parts of the body and contemplative theories may guide the formulation of such hypotheses. Future research could examine to what extent contemplative practices enhance a sense of presence or agency that can account for improvements in self-efficacy and related aspects of mental health. In particular, movement practices such as yoga and tai chi may more readily promote an increased sense of agency in which the opportunity to explore sensorimotor signals in slow, controlled movements is readily apparent.

### Increased Positive Experiences

Another downstream consequence of enhanced interoceptive capacity may be the ability to engage with and appreciate *pleasant sensations*. In the case of mindful eating interventions, for example, practices involve bringing greater attention to the pleasure of seeing, smelling, tasting, and eating of palatable foods and noticing when the satisfaction subsides, as taste-specific satiety mechanisms become apparent (Kristeller and Wolever, 2010; Daubenmier et al., 2011). Indeed, many mindfulness training programs involve a raisin-eating exercise (Kabat-Zinn, 1990), in which increased sensory precision weighting is encouraged to decenter habitual interpretations of the consumptive act, replaced with re-centering on unexpected positive aspects of the experience. The ability to enter into such sensory states and appreciate positive sensations is important, because reduced response in the brain’s reward regions to consumption of palatable foods such as milkshakes is associated with increased, unwanted weight gain (Stice et al., 2010). Such reduced activation may account for the subjective experience of “chasing the flavor” and continued eating. It remains to be determined whether increased awareness of the pleasurable taste of palatable foods increases reward activation and ultimately reduces food consumption, in particular among those who are overweight. Similarly, mindfulness-based interventions during recovery from drug addiction appear to increase interoceptive responsiveness to natural reward while decreasing responsiveness to drug cues, an effect correlated with reductions in drug craving (Garland et al., 2014). As interoceptive attention broadens to allow reactions to less conditioned cues, it appears that freedom from maladaptive cycles of craving may follow (Khanna and Greeson, 2013). Furthermore, classical accounts of mindfulness training suggest that the continued exploration of experience, including interoceptive conditioning, can itself lead to feelings of joy and rapture (Brewer et al., 2013); the importance of such feelings is made concrete in consideration of mindfulness’ impact in these and other clinical disorders.

### Embodied Effects

Finally, it is important to note that because mindfulness practice itself takes place in an embodied context, engagement with interoceptive processes may promote further physiological effects. One example common to all Buddhist traditions is the importance of sitting posture in cultivating attentional stability and emotional equipoise. As traditionally stated in the Tibetan Buddhist tradition: “When the body is straight, the channels are straight, when the channels are straight, the energies are straight, when the energies are straight the mind is straight” (Rinpoche, 1998). An upright sitting posture is thought to affect the movement of energy through the channels thereby enhancing the effectiveness of the meditation practice. One could hypothesize that just sitting in this posture without engaging in a meditative practice may have beneficial effects. Upright postures are associated with enhanced physiological outcomes in hospital patients (Convertino, 2003). And indeed, sitting in an upright posture without actually engaging in meditation practice, referred to as “sham meditation,” is associated with slowed respiration rate which predicts decreased pain unpleasantness ratings (Zeidan et al., 2010b). Another example is mindfulness meditation’s association with increased heart rate variability (HRV; Ditto et al., 2006), particularly in the high frequency band, an indicator of parasympathetic activation (Wu and Lo, 2008; Krygier et al., 2013). Greater resting HRV is associated with a host of cognitive benefits; including greater sustained attention, working memory, and motor-response control (Thayer et al., 2009). Thus, as the mindfulness practice itself unfolds, mindful attention impacts physiological systems, which in turn may feed back to influence cognitive processes in a self-reinforcing cycle. In other words, the process of attending to interoceptive sensations provides time for autonomic processes to restore homeostasis, rather than perpetuating inefficient or maladaptive regulatory habits that rely on overt behavioral intervention. Awareness of homeostasis may generate feelings of calm, peace, and satisfaction, greater connection to others, and a decreased desire to seek out externally rewarding stimuli to maintain a hedonic set point. Unlike external reward, the reward of increasingly accurate perceptual predictions may be continuous. However, this is not to say that mindfulness meditation predisposes one to passive monitoring states, but such training may increase the ability to respond more adaptively to environmental challenges and return to homeostasis more quickly.

Taken together, an interoceptive consequence of contemplative practice is the decoupling of hedonic stimulus response arcs, i.e., an increased capacity to refrain from automatically responding to aversion with avoidance and to pleasure with approach. This relative freedom from interoceptive appraisal habits that drive behavior may optimize homeostasis that becomes a self-reinforcing process. Thus, this model may serve as a working hypothesis for the mechanisms of action underlying how changes in interoceptive awareness resulting from contemplative practice may enhance health and well-being including affective and dissociative disorders, pain, and addiction. Research is needed to better understand what difference variations in the particular foci of interoceptive attention make toward promoting salutary effects.

### A Caveat on the Primacy of Interoceptive Processing

Admittedly, it seems unlikely that contemplative training can or should aim to engender continual and total awareness of the panoply of interoceptive sensations present in each moment. Instead, skillful attention to interoceptive sensation may improve self-regulation (Bornemann et al., 2014), allowing an individual to operate more closely to his or her optimal homeostatic state. We have argued that increased interoceptive attention represents one way to return to ‘being’ in a world that prioritizes ‘doing,’ restoring balance between the two. This balance may then serve to promote regulatory flexibility, with the ideal of bringing ‘flow’ to experience, the alignment of being with doing in living a meaningful and satisfying life. It may be that one reason focusing on the body has such positive potential is that rumination, already shown to be largely a negative factor in experience, operates, by and large, by shifting attention to past or future (Killingsworth and Gilbert, 2010). By contrast, the body is always in the present. Attending to the body therefore anchors the mind in the present and away from rumination. Admittedly, there are other forms of present-centered foci that would distract from rumination such as watching television, and the research literature supports many short-term regulatory benefits of distraction strategies (Sheppes and Meiran, 2007; Denson et al., 2012). However, distraction may lead to later ‘rebound effects’ when a person is faced with an unresolved stressor compared to conditions in which that stressor was directly attended to (Thiruchselvam et al., 2011). While more work is needed to properly distinguish the effects of attention-based regulatory strategies such as mindful attention and distraction, skillfully attending to the stressor itself may be the more adaptive strategy (Kross and Ayduk, 2008), and in any case an improvement over rumination.

## The Road Ahead

### Other Elements of Traditional Contemplative Approaches to Interoception

Continued dialog with representatives of traditional contemplative practices may further explore how they understand and modulate the flow of sensations through the body to enhance health and healing. From many Asian medical/contemplative perspectives, disturbances or blockages in the ‘flow’ of *lung/ch’i/prāṇa* sensations are related to disease, and free movement of *chi* is related to health, as well as to insight, kindheartedness, and other positive qualities, a central principle of Tibetan medical literature (Loizzo et al., 2009). Their understanding of the body is not only a matter of interoceptive awareness; it is most explicitly a pathway for exploring and deeply revising the sense of self, agency, and substantiality that bears on virtually every activity in life. The import of these issues, and their potential significance for mental and physical health outcomes could be a fruitful endeavor for future research.

In these Asian traditions, the interior of the body, deep in the belly, or the heart, or deep inside the head, are all significant areas for focusing the mind. The mapping of channels and the “steeds of wind” moving through them further illustrate the body’s interiority as characterized in traditional Asian or Buddhist perspectives. We also see that varying the locus of attention, for example shifting one’s focus from breath to sound, or to a particular area of the body, may produce different results.

Contemplative traditions may have theoretical insights on how placement of attention in certain parts of the body affects perception, cognitive, and emotional responses. Practices vary according to whether the body is alone or in a group, with or without direct eye contact, whether the scope of attention includes others, whether one sits in silence or performs movement, whether one attends to sound or focuses on other sensory stimuli. There is also variation in the types of intentional and cognitive framework surrounding the practice. How these variations in interoceptive training may influence cognitive, emotional, and behavioral processes could be tested in empirical research.

While the focus of this discussion has largely been on the use of contemplative practices in general, and mindfulness in particular, to increase awareness of interoceptive processes as a means to enhance self-regulation, other practices, such as those found in the Tibetan Buddhist tradition, involve the use of interoceptive awareness, sometimes in combination with mental imagery or somatic manipulations, to intentionally *modulate* the ‘flow’ of sensations through the channels. Importantly, these practices take place within specific philosophical and ethical understandings, such as believing the nature of self and all of existence to be impermanent and interdependent. From an integrated mind–body perspective, in which ‘currents flowing through the body’ are conceived as the energetic support for consciousness, such “body-oriented” practices can be a direct means to impact psychological functioning and achieve meditative states of realization. One example is the practice of *tummo,* which has been studied by scientists showing dramatic increases in core body temperature (Kozhevnikov et al., 2013). While the short-term physiological outcomes of this practice have been studied for years (Benson et al., 1982), further attention could be devoted to understanding traditional theories of how these practices work and how conscious modulation of interoceptive sensations, especially when supported by accompanying intentional body-imagery, may impact physiological and psychological functioning more generally by promoting autonomic control of physiological processes. Equally interesting in this regard would be a consideration of shifts in ‘energetic flows’ and related sensations that accompany the cultivation of love or compassion, or the use of simple sounds and their impact on mind and body. First-person descriptions can be useful here as well, since they typically will describe both physical and mental responses to such training.

### Future Research Directions

Although we have rich contemplative traditions to draw from, as well as new interoceptive awareness-enhancing approaches and emerging theory from a psychology perspective, we are facing a shortage of tools, paradigms, and appropriate measures to rigorously interrogate and integrate them. With this challenge in mind, we derive the following topics for further research from our considerations:

(1) Qualitative exploration of body-based practices from traditional contemplative traditions, and greater understanding of Asian (and eventually other) contemplative and medical models of the body that involve the ‘flow’ of ‘energetic currents’ through the body.
(2) Further refinement of scientific conceptualizations of mindfulness, including exploration of first-person accounts, to see how these match the conceptual framework for interoceptive awareness provided here. Concurrently, we must continue to explore traditional contemplative texts and practice traditions for new perspectives, and to deepen our understanding of constructs already appropriated into scientific models of interoception.
(3) Developing and/or refining appropriate quantitative measures, objective and self-report, able to capture cognitive, behavioral, and physiological changes occurring with interoceptive awareness training, moving beyond measures of interoceptive accuracy to include attention habits, sensitivity to interoceptive cues, coherence of physiological and subjective changes, and regulatory strategies.
(4) Developing research designs that integrate first, second and third-person perspectives, such as neurofeedback, in which third-person objective measures of physiological or neural change provide second-person reports to the participant in real time, potentially modulating the quality of first person experience during interoceptive attention (Lutz and Thompson, 2003).
(5) Finding appropriate ways to operationalize behavioral interventions representing mindful interoceptive awareness, e.g., to the breath.
(6) Defining clear criteria for when or under which conditions interoceptive awareness is beneficial in treating patients with psychiatric, psychosomatic, and pain conditions.
(7) Using neuroscience tools and technique to follow the process/flow of interoceptive information through the brain, e.g., by EEG oscillations or with fMRI with its top–down modulations. For example, interoceptive cultivation should be apparent in terms of increased correspondence between feedback-evoked EEG potentials and subsequent physiological change. Conversely manipulation of interoceptive signals such as respiratory occlusion following enhanced interoception should provoke stronger and more reliable evoked potentials.
(8) Develop longitudinal studies applying interoceptive awareness training to patients with clinical conditions.
(9) Consider testing effects of mindfulness training and other contemplative practices on existing measures of presence and agency (rubber hand illusion, virtual environment experiments) to see if they mediate improvements in health outcomes.

By improving the measurement of interoception at subjective and physiological levels, and seeking to better understand the coherence (and lack thereof) between these levels of representation, it may be possible to broaden our understanding of interoception and its connection to motivation and well-being. Furthermore, such paradigms will help to test basic properties of information processing in the brain, investigating how we both predict and adjust to changing sensory stimuli, and how such stimuli are incorporated into a broader motivational milieu.

### Concluding Remarks

Interoception has in many ways been a hidden sense, perhaps due to the challenges involved in measuring and manipulating interoceptive signals. And yet, interoception is arguably at least as important as the external senses, providing a sense of embodiment in the world that is foundational to a person’s subjective sense of well-being. Contemporary scientific frameworks largely lack the ability to articulate contemplative models of how energy within the body helps to determine well-being, but, concepts such as the simulation map offer an avenue for bridging this cultural divide. Indeed, the idea of energy or prana may one day be expressed as the allocation of metabolic and cognitive resources to afford neural representation of body states. While genetic, social, and environmental factors clearly have great impact on a person’s quality of life, our goal in writing this paper is to bring to light the process by which health and suffering are revealed to us in the moment, in the hope of providing a more integrated psychological account.

Fortunately, interest in interoception appears to be growing, and its researchers are privileged to have access to a rich repository of conceptual models from neuroscience, clinical, and contemplative research traditions. The challenge for the field will be to pay careful attention to the claims made in each of these traditions, to better characterize their predictions, and in doing so to more even-handedly advance modern secular interoceptive science while respecting the validity of all of these perspectives. While perhaps no single researcher will hold sufficient expertise in all of these domains, through collaboration such integration is possible- we hope that the current paper and other articles contained in the special issue is testament to that lofty but achievable goal. It is our hope that we may all benefit from the continued study of interoception, in paralleling the advances modern science has already made in understanding the external world.

## Conflict of Interest Statement

The authors declare that the research was conducted in the absence of any commercial or financial relationships that could be construed as a potential conflict of interest.
